# Anti-IL-20 Antibody Protects against Ischemia/Reperfusion-Impaired Myocardial Function through Modulation of Oxidative Injuries, Inflammation and Cardiac Remodeling

**DOI:** 10.3390/antiox10020275

**Published:** 2021-02-10

**Authors:** Kun-Ling Tsai, Wan-Ching Chou, Hui-Ching Cheng, Yu-Ting Huang, Ming-Shi Chang, Shih-Hung Chan

**Affiliations:** 1 Department of Physical Therapy, College of Medicine, National Cheng Kung University, Tainan 701, Taiwan; kunlingtsai@mail.ncku.edu.tw (K.-L.T.); jinjaychou@gmail.com (W.-C.C.); qaz200408@gmail.com (H.-C.C.); 530.yuting@gmail.com (Y.-T.H.); 2 Institute of Allied Health Sciences, College of Medicine, National Cheng Kung University, Tainan 701, Taiwan; 3 Institute of Basic Medical Sciences, College of Medicine, National Cheng Kung University, Tainan 701, Taiwan; 4 Department of Biochemistry and Molecular Biology, College of Medicine, National Cheng Kung University, Tainan 701, Taiwan; 5 Department of Internal Medicine, National Cheng Kung University Hospital, College of Medicine, National Cheng Kung University, Tainan 701, Taiwan

**Keywords:** acute myocardial infarction (AMI), ischemia/reperfusion (I/R) injury, oxidative stress, cardiac remodeling

## Abstract

Acute myocardial infarction (AMI) is the most critical event in the disease spectrum of coronary artery disease. To rescue cardiomyocytes in AMI, it is important to restore blood supply as soon as possible to reduce ischemia-induced injury. However, worse damage can occur during the reperfusion phase, called the reperfusion injury. Under ischemia/reperfusion (I/R) injury, elevated oxidative stress plays a critical role in regulation of apoptosis, inflammation and remodeling of myocardium. Our previous study has demonstrated that interleukin (IL)-20 is increased during hypoxia/reoxygenation stimulation and promotes apoptosis in cardiomyocytes. This study was, therefore, designed to investigate whether IL-20 antibody could reduce I/R-induced myocardial dysfunction. Results from this study revealed that IL-20 antibody treatment significantly suppressed I/R-induced nicotinamide adenine dinucleotide phosphate oxidase, oxidative stress, apoptosis, proinflammatory responses, cardiac fibrosis, and expression of cardiac remodeling markers in Sprague-Dawley rats. Plasma B-type natriuretic peptide level was also reduced by IL-20 antibody injection. IL-20 antibody treatment appeared to restore cardiac function under the I/R injury in terms of greater values of ejection fraction and fractional shortening compared to the control group. Two commonly used indicators of cardiac injury, lactate dehydrogenase and creatine kinase-MB, were also lower in the IL-20 antibody injection group. Taken together, our results suggested that IL-20 antibody holds the potential to reduce the I/R-elicited cardiac dysfunction by preventing cardiac remodeling.

## 1. Introduction

Acute myocardial infarction (AMI) is a life-threatening disease. Patients may die before hospital arrival. Even though patients can survive till hospital admission, mortality and morbidity are still high [1]. AMI may lead to impaired cardiac contractility and congestive heart failure (CHF) [2]. Patients with CHF have higher mortality and morbidity as well as worse quality of life than the normal population [3]. AMI is the most critical event in the disease spectrum of coronary artery disease, distinguished by endothelial damage, lipid aggregation, and the generation of atherosclerotic plaques in the vessel wall of coronary artery [4]. Atherosclerosis-caused coronary artery luminal obstruction and plaque rupture are the most common issues in the setting of AMI [5]. In order to rescue cardiomyocytes in AMI, it is important to restore blood supply as soon as possible. However, reperfusion can provoke additional damage to ischemic tissue, the so-called ischemia/reperfusion (I/R) injury [6].

The return of blood flow to the ischemic areas causes high amount of reactive oxygen species (ROS) production to trigger rapid and critical injury to cardiomyocytes [7]. Origins of ROS in myocardial reperfusion comprise the superior activation of nicotinamide adenine dinucleotide phosphate (NADPH) oxidases, which are expressed in different cell types in myocardial tissue [8]. NADPH oxidase-2 (NOX-2) is one of the major regulators of O_2_^−^ and H_2_O_2_ formation in the heart. In addition, NOX-2 plays a critical role in the modulation of growth and death in cardiomyocytes. In response to I/R injuries, NOX-2 is activated to induce ROS generation and consequently myocardial damage [9].

NADPH oxidases that catalyze the generation of free radicals are the main origins of ROS in cardiomyocytes during I/R [10]. The activation of NADPH oxidases causes oxidative injuries and left ventricular dysfunction in part because of mitochondrial insufficiency induced by elevated O_2_^−^ generation, mitochondrial dysfunction, and cardiac apoptosis [11]. Apoptosis and necrosis of cardiomyocytes with subsequent extravagant inflammation are the main causes of cardiomyocyte damage in AMI [12]. Cell death during AMI induces a multiphase reparative response in which the damaged tissues are replaced with fibrotic scars. This is followed by remodeling of the surrounding myocardium, and eventually impaired cardiac function and resultant CHF develop [13].

Proinflammatory cytokines secreted from cardiomyocytes after hypoxia or ischemia stimulation can elicit additional cellular inflammatory responses, and subsequent cytotoxic injury [14]. For example, interleukin (IL)-6 and IL-6 related proinflammatory cytokines released by cardiomyocytes are critical in the modulation of cardiac apoptosis and hypertrophy [15]. In addition, in the circumstances of I/R, signaling transduction pathways of cardiomyocytes converge on the activation of mitogen-activated protein kinases (MAPKs) and nuclear factor kappa-light-chain-enhancer of activated B cells (NF-κB), thereby triggering proinflammatory responses and transforming growth factor beta (TGF-β) signaling. TGF-β1 had been reported as a critical switch controlling the transformation from inflammation to fibrosis in the late phase of myocardial infarction [16]. Thus, taken together, the strategies designed to protect cardiomyocytes from death and manage unsuitable proinflammatory response are required for clinical improvement in patients with AMI.

IL-20, a member of the IL-10 family of cytokines, was discovered in 2001. IL-20 acts on multiple cell types by activating a heterodimer receptor complex of either IL-20R1–IL-20R2 or IL-22R1–IL-20R2. There have been several lines of evidence indicating that the interaction of IL-20 with its receptors might have proinflammatory effects on chronic inflammatory diseases, particularly rheumatoid arthritis, osteoporosis, and breast cancer [17]. Our previous study had concluded that IL-20 is responsive to hypoxia/reoxygenation stimulation in vitro and in rat hearts undergoing I/R injury. We reported that IL-20 elicited an increase in Ca^2+^ and activation of the protein kinase C and NADPH oxidase pathway, leading to the elevation of oxidase stress and downregulation of protein kinase B, also known as AKT [18]. In this study, we hypothesized that IL-20 antibody is an effective treatment option for reducing I/R-induced oxidative stress, apoptosis and inflammation, thereby mitigating cardiac fibrosis and cardiac dysfunction.

## 2. Materials and Methods

### 2.1. Cell Culture and Reagents

H9C2 cells, which are myoblast cells from rat myocardium, were purchased from the American Type Culture Collection(Manassas, VA, USA). H9C2 cells were cultured in Dulbecco’s modified Eagle’s medium (DMEM) supplemented with 10% fetal bovine serum (FBS) and penicillin (50 IU/mL)/streptomycin (50 μg/mL). A 0.25% (*w/v*) Trypsin-0.53 mM ethylenediaminetetraacetic acid (EDTA) solution was used to passage cells. Cells were cultured in humidified air with 5% CO_2_ at 37 °C. FBS and EDTA were purchased from Thermo Fisher Scientific (Waltham, MA, USA). Diphenyleneiodonium chloride (DPI), SB203580, Glutathione (GSH), and JSH-23, were purchased from MilliporeSigma (St. Louis, MO, USA). The cell-permeant 2′,7′-dichlorodihydrofluorescein diacetate (H2DCFDA) was obtained from Thermo Fisher Scientific (Waltham, MA, USA).

### 2.2. Hypoxia/Reoxygenation (H/R)

H9C2 cells were washed two times with PBS to remove glucose and serum in the culture medium. In control cells, the medium was replaced with glucose-free DMEM; in IL-20 antibody-treated or inhibitor-coincubated cells, the medium was replaced with IL-20 antibody or relevant inhibitors containing glucose-free DMEM. The cells were transferred to a hypoxia chamber containing of 95% N_2_ and 5% CO_2_ for 1 h. After hypoxia exposure, the cells were placed in a 5% CO_2_ and 95% O_2_ incubator for 4 h reoxygenation with high-glucose DMEM containing of 10% FBS. In IL-20 silencing cells, cells were transfected with IL-20 siRNA for 48 h before exposure to H/R.

### 2.3. Antibody Preparation

Anti-IL-20 monoclonal antibody was generated with standard protocols as described in our previous study [19]. Our anti-IL-20 monoclonal antibody has been well demonstrated to specifically repress and neutralize the biological function of IL-20 in vitro and in vivo [20,21].

### 2.4. Animal Model for Ischemia-Reperfusion (I/R) Injury

The animals in this study received humane treatment. In agreement with the 3Rs principle of reduction, the experimental animals were already scheduled for investigations on I/R injury. A total of 18 healthy male Sprague-Dawley (SD) rats (200–250 g, 8–9 weeks old) were bought from BioLASCO Taiwan. Animals were housed in the temperature-controlled room (21–22 °C) and fed with regular food in the Laboratory Animal Center, College of Medicine, National Cheng Kung University. The animals were kept in groups of 2–3 animals in cages in the laboratory animal center for at least 7 days after arrival. The diurnal rhythm was regulated with 12 h light and 12 h dark. The SD rats were randomly numbered and assigned to three groups. For ischemia induction in animals, the rats were anesthetized intramuscularly using the mixture of 10:1 tiletamine/zolazepam (Zoletil) (Virbac, Carros, France) and xylazine (Rompun) (Bayer, Pittsburgh, PA, USA). The dosage of anesthesia was 0.1 mL Zoletil/100 g body weight. Next, the heart was accessed by left thoracotomy, and the pericardium was removed. Ischemia was induced via the ligation of the left anterior descending coronary artery (LAD) with a 6-0 silk suture. After 1 h, ligation of LAD was released to allow reperfusion. For the IL-20 antibody treatment group, IL-20 antibody (5 mg/kg) was injected at the moment of reperfusion via intraperitoneal injection. This dosage was based on our previous study [20]. The animals in the control group underwent the same surgical procedures but without LAD ligation. At the time of closure, the antibiotic (neomycin powder) was applied onto the surgical wound. The Bactermin Ointment was applied on the skin for preventing infection. The wound of thoracotomy was dressed daily to prevent any infection and to monitor for any dehiscence of the suture area. Two analgesic drugs were used after thoracotomy (Nalbuphine, 6 mg/kg, every 4–6 h; Ketoprofen, 2 mg/kg, every 24 h). For reducing anxiety or stress after operation, the cages were enriched with paper. The food pellets were put on the bottom the cages to promote access. Seven days after surgery, the animals were sacrificed for further experiments. The sacrifice was conducted by injection of overdose Zoletil and Rompun intramuscularly and then inhalation of overdose isoflurane. This animal study was approved by the Institutional Animal Care and Use Committee (IACUC) of National Cheng Kung University (IACUC No. 107179).

### 2.5. Extraction of Proteins from Tissues, Western Blotting Assay, and Plasma Preparation

Protein expression levels were investigated by Western blotting. Total protein was isolated from cells of the left ventricle. After sacrifice, the hearts of the animals were collected. The tissue of the left ventricle was washed two times with phosphate-buffered saline (PBS), and then 100 mg of tissue was cut for homogenization with radioimmunoprecipitation assay (RIPA) lysis buffer. The homogenates were centrifuged at 13,000× *g* for 30 min, and the supernatant was collected and placed at −80 °C until use. For Western blotting, proteins were transferred to a polyvinylidene difluoride membrane after separation by electrophoresis on sodium dodecyl sulfate polyacrylamide gels. The membranes were blocked by the blocking buffer for 1 h at 37 °C and incubated with primary antibodies for 18 h at 4 °C followed by hybridization with horseradish peroxidase-conjugated secondary antibodies for 1 h. The intensities of protein bands were quantified by densitometric analysis. Plasma was obtained, on the day of sacrifice, through blood collection for the measurement of malondialdehyde (MDA), IL-8, superoxide dismutase (SOD) activity, lactate dehydrogenase (LDH), creatine kinase-MB (CK-MB) assay, and B-type natriuretic peptide (BNP). For in vitro investigations, cells were collected in tubes, RIPA lysis buffer was used for protein isolation. NF-κB p65 Transcription Factor Assay Kit (ab133112) and NADP/NADPH Assay Kit (ab65349) were obtained from Abcam (Cambridge, MA, USA).

### 2.6. Antibodies

Anti-NOX-2, anti-Rac-1, anti-p47phox, anti-p-53, anti-Bax, anti-Bcl-2, anti-cytochrome c, anti-β-actin, anti-p-I-κBα, anti-p-p38, anti-p-NF-κB, anti-COX-2, anti-IL-8, anti-TGFβ1, anti-p-ERK, anti-Sp1, anti-CTGF, anti-FGF2, anti-uPA, anti-MMP-2, anti-MMP-9, and anti-α-SMA were purchased from Thermo Fisher Scientific (Waltham, MA, USA). Secondary antibodies were obtained from Cell Signaling (Danvers, MA, USA).

### 2.7. Isolation of mRNA and Quantitative Real-Time Polymerase Chain Reaction (PCR)

Total RNA was isolated from H9C2 cells using the RNeasy kit (Qiagen, Valencia, CA, USA). Oligonucleotides were designed using the computer software package Primer Express 2.0 (Applied Biosystems, Foster City, CA, USA). All of the oligonucleotides were synthesized by Invitrogen (Breda, The Netherlands). Oligonucleotide specificity was determined by a homology search within the genome (BLAST, National Center for Biotechnology Information, Bethesda, MD, USA) and confirmed by dissociation curve analysis. The oligonucleotide sequences are provided in the Supplementary Table. PCR was performed with SYBR Green in an ABI 7000 sequence detection system (Applied Biosystems) according to the manufacturer’s guidelines.

### 2.8. Enzyme-Linked Immunosorbent Assay (ELISA) and Antioxidant Enzyme Activity Assay

ELISA was performed using commercial kits according to the manufacturer’s instructions. In brief, the antibody in the coating buffer was added to individual wells and incubated for 2 h at 37 °C. After incubation, the coating solution was removed, and wells were washed with PBS-0.05% Tween-20 twice. Then, 100 μL blocking buffer was loaded in each well for 1 h at 37 °C. After blocking, wells were washed with PBS-0.05% Tween-20 twice. An aliquot of 50 µL of diluted antibody was added to each well for 1 h of incubation. Next, 50 µL of conjugated secondary antibody was added to each well for 1 h of incubation. The absorbance wavelength was set at 450 nm. The IL-8 kit was bought from R&D (Minneapolis, MN, USA). The BNP and MDA kits were bought from Abcam (Cambridge, MA, USA). The kits for CK-MB, LDH, and SOD activity were purchased from Biovision (San Francisco, CA, USA).

### 2.9. Determination of Cardiac Functional Parameters

Four days after operation, echocardiography was performed to evaluate cardiac function. Isoflurane-anesthetized animals were placed in a supine position. Echocardiographic data were collected by a Vevo 770 microimaging system with a 25-MHz probe (VisualSonics, Toronto, ON, Canada). Parameter values were collected based on the M-mode and two-dimensional images obtained in the parasternal long and short axis views at the level of the papillary muscles.

### 2.10. Apoptotic Assay

For investigating apoptosis in animal cardiac tissues, the terminal deoxynucleotidyl transferase dUTP nick end labeling (TUNEL) assay was performed. Tissues were soaked in 4% paraformaldehyde. Then, paraffin-embedded myocardium was cut into 2-μm-thick sections. These tissue sections were deparaffinized in xylene, rehydrated through a graded alcohol series (100%, 90%, 85%, and 75%), and then rinsed in PBS (pH 7.2). TUNEL-based DNA fragmentation detection kit (FragEL; Calbiochem, San Diego, CA, USA) was used to detect apoptotic cells in cardiac tissue sections. Apoptosis positive cells in H9C2 were studied by the flow cytometry.

### 2.11. Masson’s Trichrome Staining

Masson’s trichrome staining was used for the investigation of histologically fibrotic changes using a Trichrome Stain (Masson) Kit from MilliporeSigma (St. Louis, MO, USA). All procedures were performed according to the manufacturer’s instructions. The total ventricular area and the area of fibrotic changes were assigned numerical values, and the fibrotic changes were normalized by the left ventricle.

### 2.12. Measurement of ROS Production

The production of ROS in H9C2 cells was determined by H2DCFDA. Confluent H9C2 cells (10^4^ cells/well) in 96-well plates were exposed to H/R. After removing medium from the wells, the cells were incubated with 10 μM H2DCFDA for 1 h. The fluorescence intensity was measured with the Fluoroskan™ microplate fluorometer from Thermo Fisher Scientific (Waltham, MA, USA) calibrated with excitation at 540 nm and emission at 590 nm. The percentage increase in fluorescence per well was calculated by the formula [(Ft2 − Ft0)/Ft0] × 100.

### 2.13. Statistical Analysis

Statistical analysis was performed with Statistical Product and Service Solutions (SPSS) software version 11.0 (SPSS, Inc., Chicago, IL, USA). Results are expressed as the means ± standard deviation (SD). Two-group comparisons were analyzed by a two-sided Student’s *t*-test. One-way analysis of variance was used to compare data among groups in experiments including three or more groups. A *p*-value < 0.05 was considered statistically significant.

## 3. Results

### 3.1. IL-20 Antibody Reduces I/R-Induced Oxidative Damages by Modulating Expression of NADPH Oxidase Subunits

**Figure 1:**
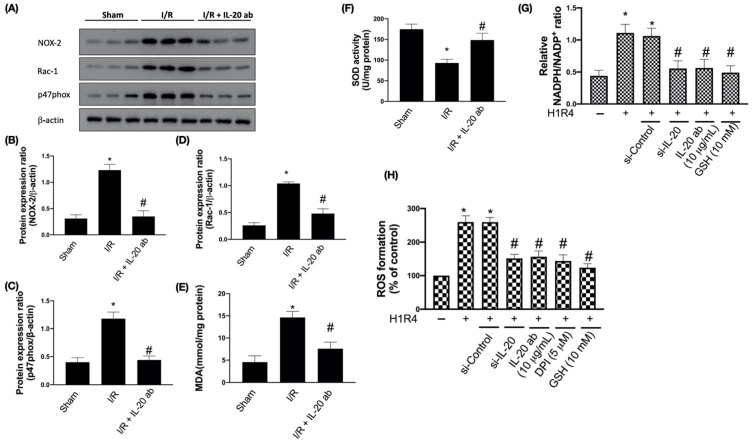
Interleukin (IL)-20 antibody reduces ischemia/reperfusion (I/R)-induced oxidative stress through nicotinamide adenine dinucleotide phosphate (NADPH) oxidase inhibition. Sprague Dawley rats receiving sham operation or ischemia/reperfusion (I/R) treatment were studied. In the IL-20 antibody treatment group, animals were injected with 5 mg/kg IL-20 antibody at the phase of reperfusion. The left ventricular tissues were collected for Western blotting assay. Representatives of Western blot (**A**), and the densitometric analysis of NADPH oxidase-2 (NOX-2) (**B**), p47phox (**C**), and Rac-1 (**D**) were shown. Plasma malondialdehyde (MDA) concentration (**E**) and superoxide dismutase (SOD) activity (**F**) were checked. The data were presented as the mean ± standard deviation (SD) of six animals in each group. (**G**) NADPH oxidase activity in H9C2 cells after hypoxia/reoxygenation (H/R) was presented by NAPDH/NADP^+^ ratio. H9C2 cells that had been first exposed to hypoxia chamber with oxygen-glucose deprivation (OGD) for 1 h were moved into the normoxia chamber with high glucose medium for further 4 h, a condition abbreviated as H1R4. In some cases, cells were transfected with si-Control or si-IL-20 for 48 h before OGD. In the IL-20 antibody treatment group, 10 μg/mL IL-20 antibody was added to medium during H/R. Glutathione (GSH), an antioxidant, was used for mitigating oxidative stress. (**H**) Reactive oxygen species (ROS) concentration was investigated by the cell-permeant 2′,7′-dichlorodihydrofluorescein diacetate. Diphenyleneiodonium chloride (DPI), an inhibitor of NADPH oxidase, was used to inhibit H/R-induced NADPH oxidase activation. Data were presented as the mean ± SD of three different experiments. (* indicating *p* < 0.05 compared to the sham group or normoxia control cells; # indicating *p* < 0.05 compared to the I/R group or H/R cells).

NADPH oxidase plays a critical role in causing oxidative stress and myocardial damage after I/R. Inhibition the activity of NADPH oxidase can effectively repress excessive formation of ROS [22]. We found that I/R enhanced NOX-2, Rac-1 and p47phox levels in cardiac tissues, whereas this effect could be blocked by IL-20 antibody (5 mg/kg) (Figure 1A–D). In order to examine whether IL-20 antibody treatment can reduce I/R-induced oxidative stress, we measured MDA level, a marker of cardiac oxidative damage, and SOD activity from plasma. Injection of IL-20 antibody after reperfusion significantly reversed the level of MDA (Figure 1E) and upregulated the activity of SOD compared to that of control I/R animals (Figure 1F). Results from Figure 1A–F indicated that IL-20 antibody protects against I/R-induced oxidative stress, which might be through modulation of NADPH oxidase activity. In order to confirm this issue, we conducted in vitro investigations using H/R intervention in H9C2 cells. We found that both silencing IL-20 expression and IL-20 antibody coincubation reduced H/R-caused elevation of NAPDH/NADP^+^ ratio (Figure 1G) and ROS concentrations (Figure 1H). GSH (an antioxidant) coincubation mitigated H/R-caused elevation of NAPDH/NADP^+^ ratio and ROS level (Figure 1G,H). DPI (an inhibitor of NADPH oxidase) coincubation also inhibited H/R-increased ROS level (Figure 1H).

### 3.2. IL-20 Antibody Mitigates I/R-Caused Cardiac Apoptosis via Modulation of the Mitochondria-Dependent Pathway

**Figure 2:**
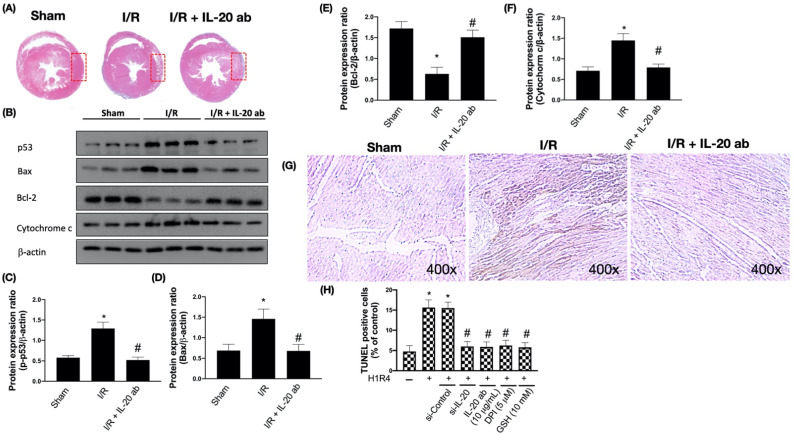
Interleukin (IL)-20 antibody reduces ischemia/reperfusion (I/R)-induced apoptosis by modulation of the mitochondria-dependent apoptosis pathways. Sprague Dawley rats receiving sham operation or I/R treatment were studied. In the IL-20 antibody treatment group, animals were injected with 5 mg/kg IL-20 antibody at the phase of reperfusion. (**A**) Histological assessment of infarct area after I/R was shown. Tissue sections were obtained from formalin-fixed heart slices that were treated with hematoxylin and eosin staining. The left ventricular tissues were collected for Western blotting assay. Representatives of Western blot (**B**), and the densitometric analysis of p-53 (**C**), Bax (**D**), Bcl-2 (**E**), and cytochrome c (**F**) were shown. (**G**) Apoptosis in tissue was analyzed by terminal deoxynucleotidyl transferase dUTP nick end labeling (TUNEL) assay. (**H**) Apoptosis in H9C2 cells after hypoxia/reoxygenation (H/R) was presented by TUNEL assay using flow cytometry. H9C2 cells that had been first exposed to hypoxia chamber with oxygen-glucose deprivation (OGD) for 1 h were moved into the normoxia chamber with high glucose medium for further 4 h, a condition abbreviated as H1R4. In some cases, cells were transfected with si-Control or si-IL-20 for 48 h before OGD. In the IL-20 antibody treatment group, 10 μg/mL IL-20 antibody was added to medium during H/R. Glutathione (GSH), an antioxidant, was used for mitigating oxidative stress. Diphenyleneiodonium chloride (DPI), an inhibitor of nicotinamide adenine dinucleotide phosphate (NADPH) oxidase, was used to inhibit H/R-induced NADPH oxidase activation. Data were presented as the mean ± standard deviation of three different experiments. (* indicating *p* < 0.05 compared to the sham group or normoxia control cells; # indicating *p* < 0.05 compared to the I/R group or H/R cells).

Increased apoptosis is reported in the heart tissue of subjects with myocardial infarction [23]. We found that IL-20 antibody reduced I/R-caused cardiac damage by hematoxylin and eosin staining (Figure 2A). Moreover, the tumor suppressor p53 plays a central role in cellular responses to DNA damage. The activation of p53 results in cell cycle arrest as well as apoptosis [24]. We observed that I/R upregulated the expression of p53 (Figure 2B,C), whereas IL-20 antibody intervention reversed this phenomenon. Mitochondria have a critical role in the execution of apoptosis in cardiac tissues. These organelles are the main target for ROS-induced oxidative injuries [25]. Previous reports have shown increased cardiac apoptosis after myocardial I/R [26]. Hence, we investigated whether IL-20 antibody protects against I/R-caused apoptosis. Our results revealed that I/R decreased Bcl-2 expression and increased Bax and cytochrome c levels. This effect could be reversed by IL-20 antibody injection at reperfusion (Figure 2B,D–F). It has been previously reported that DNA fragmentation, a well-known marker of apoptosis, was not found in the myocardium subjected to ischemia only, but occurred during ischemia plus reperfusion [27]. The TUNEL assay results confirmed that IL-20 antibody injection at reperfusion phase reduces I/R-induced apoptosis in the myocardium (Figure 2G). IL-20 silencing, IL-20 antibody coincubation, DPI, and GSH all reduced H/R-facilitated apoptosis in H9C2 cells (Figure 2H).

### 3.3. IL-20 Antibody Reduces I/R-Mediated Activation of Proinflammatory Responses

**Figure 3:**
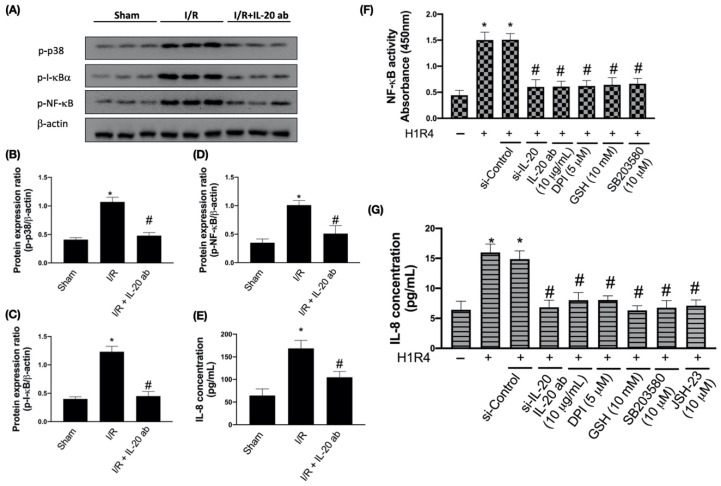
Interleukin (IL)-20 antibody mitigates ischemia/reperfusion (I/R)-induced inflammation by modulating nuclear factor kappa-light-chain-enhancer of activated B cells (NF-κB) activity. Sprague Dawley rats receiving sham operation or I/R treatment were studied. In the IL-20 antibody treatment group, animals were injected with 5 mg/kg IL-20 antibody at the phase of reperfusion. The left ventricular tissues were collected for Western blotting assay. Representatives of Western blot (**A**), and the densitometric analysis of p-p38 (**B**), p-inhibitor of NF-κB (I-κB) (**C**), and p-NF-κB (**D**) were shown. The enzyme-linked immunosorbent assay was used for the investigation of IL-8 concentration in plasma (**E**). The data were presented as the mean ± standard deviation (SD) of six animals in each group. NF-κB activity (**F**) and IL-8 release (**G**) in H9C2 cells after hypoxia/reoxygenation (H/R) intervention were checked. H9C2 cells that had been first exposed to hypoxia chamber with oxygen-glucose deprivation (OGD) for 1 h were moved into the normoxia chamber with high glucose medium for further 4 h, a condition abbreviated as H1R4. In some cases, cells were transfected with si-Control or si-IL-20 for 48 h before OGD. In the IL-20 antibody treatment group, 10 μg/mL IL-20 antibody was added to medium during H/R. Glutathione (GSH), an antioxidant, was used for mitigating oxidative stress. In some cases, diphenyleneiodonium chloride (DPI, a nicotinamide adenine dinucleotide phosphate oxidase inhibitor), SB203580 (a p38 inhibitor), or JSH-23 (a NF-κB inhibitor) was coincubated during H/R. Data were presented as the mean ± SD of three different experiments. (* indicating *p* < 0.05 compared to the sham group or normoxia control cells; # indicating *p* < 0.05 compared to the I/R group or H/R cells).

I/R upregulates the MAPK cascade and also induces the phosphorylation of I-κB kinase, thereby promoting NF-κB p65 and p50 nuclear translocation and triggering proinflammatory events [16]. We found that I/R enhanced the phosphorylation of p38, I-κBα, and NF-κB, which could be abrogated by IL-20 antibody intervention (Figure 3A–D). In addition, we also observed that I/R increased the plasma level of IL-8, an important molecule associated with cardiac ischemia injuries [28]. IL-20 antibody injection effectively attenuated I/R-mediated upregulation of IL-8 (Figure 3E). In order to confirm whether IL-20 antibody reduces I/R-promoted proinflammatory responses through inhibition of p38MAPK and NF-κB pathway, we exposed H9C2 cells to H/R and checked the activity of NF-κB and IL-8 levels with IL-20 silencing, IL-20 antibody and relevant inhibitors treatment. In H/R-treated cells, the activity of NF-κB was reduced by IL-20 silencing, IL-20 antibody treatment, DPI, GSH, and SB203580 (an inhibitor of p38MAPK) (Figure 3F). Moreover, inhibition of IL-20, NADPH oxidase, p38MAPK, and NF-κB mitigated H/R-increased IL-8 levels (Figure 3G).

### 3.4. IL-20 Antibody Attenuates Activation of I/R-Associated Fibrotic Factors

**Figure 4:**
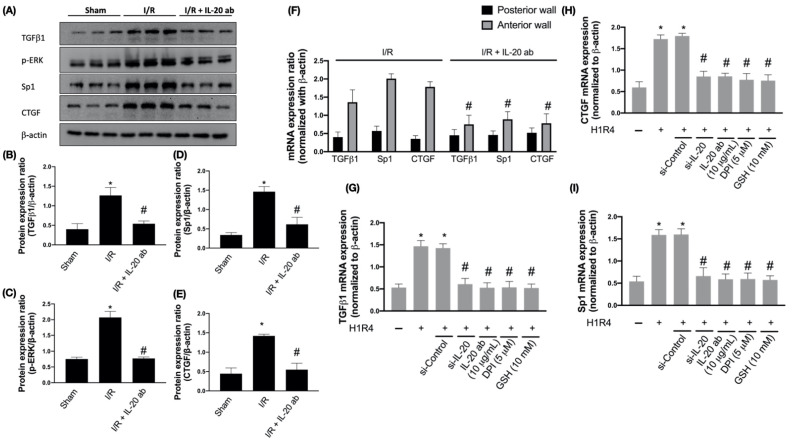
The effect of interleukin (IL)-20 antibody on ischemia/reperfusion (I/R)-induced upregulation of fibrosis factors. Sprague Dawley rats receiving sham operation or I/R treatment were studied. In the IL-20 antibody treatment group, animals were injected with 5 mg/kg IL-20 antibody at the phase of reperfusion. The left ventricular tissues were collected for Western blotting assay. Representatives of Western blot (**A**), and the densitometric analysis of transforming growth factor beta 1 (TGFβ1) (**B**), p-extracellular regulated protein kinases (ERK) (**C**), transcription factor specificity protein 1 (Sp1) (**D**), and connective tissue growth factor (CTGF) (**E**) were shown. (**F**) The mRNA levels of fibrosis factors in ischemic area (anterior ventricular wall) and nonischemic area (posterior ventricular wall) were investigated by quantitative real-time polymerase chain reaction assays. The data were presented as the mean ± standard deviation (SD) of six animals in each group. (**G**–**I**) The mRNA expression of fibrosis factors in H9C2 cells after hypoxia/reoxygenation (H/R) was examined. H9C2 cells that had been first exposed to hypoxia chamber with oxygen-glucose deprivation (OGD) for 1 h were moved into the normoxia chamber with high glucose medium for further 4 h, a condition abbreviated as H1R4. In some cases, cells were transfected with si-Control or si-IL-20 for 48 h before OGD. In the IL-20 antibody treatment group, 10 μg/mL IL-20 antibody was added to medium during H/R. Glutathione (GSH), an antioxidant, was used for mitigating oxidative stress. Diphenyleneiodonium chloride (DPI), a nicotinamide adenine dinucleotide phosphate (NADPH) oxidase inhibitor, was used to inhibit H/R-induced NADPH oxidase activation. Data were presented as the mean ± SD of three different experiments. (* indicating *p* < 0.05 compared to the sham group or normoxia control cells; # indicating *p* < 0.05 compared to the I/R group or H/R cells).

Fibrosis that ensues tissue damage is a critical part of restoration and is often associated with inflammation. Advanced fibrosis is considered a pathological feature and leads to organ dysfunction [29]. TGF-β is an important profibrotic cytokine and plays an integral role in regulating hypertrophic cardiomyocyte growth after AMI [30]. A previous study suggested that TGF-β1 is able to increase the phosphorylation of extracellular signal-regulated kinase (ERK) in fibroblasts and that phosphorylation of ERK is necessary for TGF-β1-related epithelial-to-mesenchymal transformation, a critical step for pathologic fibrosis [31]. Our data revealed that I/R enhanced the expression of TGF-β1 and phosphorylation of ERK and that IL-20 antibody injection prevented this phenomenon (Figure 4A–C). Since the transcription factor specificity protein 1 (Sp1) is involved in TGF-β1-stimulated alpha 2(I)-collagen transcription, and connective tissue growth factor (CTGF) participates in the regulation of cardiac fibrosis and heart failure [32], we examined the induction of SP1 and CTGF by I/R injury. The results show that expression levels of these two factors were increased after I/R, but the induction could be attenuated by IL-20 antibody injection (Figure 4A,D,E). Moreover, we examined the expression levels of fibrosis factors in anterior ventricular wall (ischemic area) and posterior ventricular wall (nonischemic area). We found that the expression levels of fibrosis factors were upregulated in anterior ventricular wall and IL-20 antibody treatment reduced the expression levels of fibrosis factors compared to I/R animals without IL-20 antibody treatment (Figure 4F). Furthermore, IL-20 silencing, IL-20 antibody treatment, DPI, and GSH mitigated H/R-promoted mRNA expression levels of TGFβ1, Sp1, and CTGF in H9C2 cells (Figure 4G–I).

### 3.5. IL-20 Antibody Diminishes the I/R-Caused Myocardial Remodeling-Associated Molecule Expression and Cardiac Fibrosis

**Figure 5:**
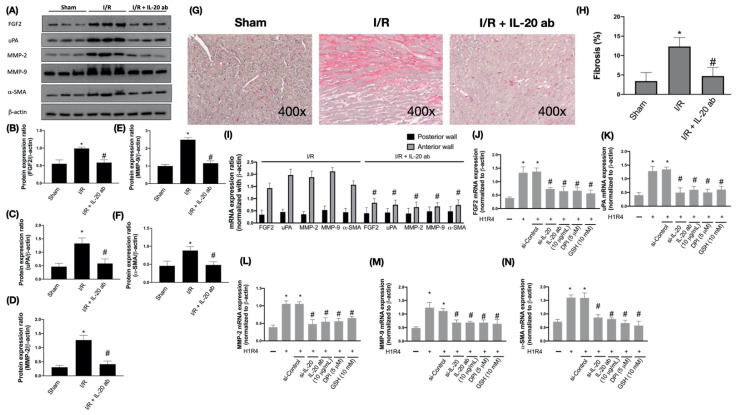
The effect of interleukin (IL)-20 antibody on ischemia/reperfusion (I/R)-induced upregulation of cardiac remodeling-related factors and fibrosis. Sprague Dawley rats receiving sham operation or I/R treatment were studied. In the IL-20 antibody treatment group, animals were injected with 5 mg/kg IL-20 antibody at the phase of reperfusion. The left ventricular tissues were collected for Western blotting assay. Representatives of Western blot (**A**), and the densitometric analysis of fibroblast growth factor 2 (FGF2) (**B**), urokinase-type plasminogen activator (uPA) (**C**), matrix metallopoateinase-2 (MMP-2) (**D**), matrix metallopoateinase-9 (MMP-9) (**E**), and α-smooth muscle actin (α-SMA) (**F**) were shown. Masson’s trichrome staining was done to detect fibrotic tissue. Representative images of Masson’s Trichrome staining (**G**) and quantification (**H**) of fibrosis areas were shown. (**I**) The mRNA levels of remodeling-related factors in ischemic area (anterior ventricular wall) and nonischemic area (posterior ventricular wall) were investigated by quantitative real-time polymerase chain reaction assays. The data were presented as the mean ± standard deviation (SD) of six animals in each group. (**J**–**N**) The mRNA expression of fibrosis factors in H9C2 cells after H/R was examined. H9C2 cells that had been first exposed to hypoxia chamber with oxygen-glucose deprivation (OGD) for 1 h were moved into the normoxia chamber with high glucose medium for further 4 h, a condition abbreviated as H1R4. In some cases, cells were transfected with si-Control or si-IL-20 for 48 h before OGD. In the IL-20 antibody treatment group, 10 μg/mL IL-20 antibody was added to medium during hypoxia/reoxygenation (H/R). Glutathione (GSH), an antioxidant, was used for mitigating oxidative stress. Diphenyleneiodonium chloride (DPI), a nicotinamide adenine dinucleotide phosphate (NADPH) oxidase inhibitor, was used to inhibit H/R-induced NADPH oxidase activation. Data were presented as the mean ± SD of three different experiments. (* indicating *p* < 0.05 compared to the sham group or normoxia control cells; # indicating *p* < 0.05 compared to the I/R group or H/R cells).

To further study the protective effect of IL-20 antibody on I/R-induced cardiac remodeling, we assessed levels of molecules involved in the progress of cardiac remodeling. Fibroblast growth factor 2 (FGF-2), the proteolytic enzymes urokinase-type plasminogen activator (uPA), and matrix metalloproteinases (MMPs) have been reported to play an important role in adverse cardiac remodeling [33,34]. Our results show that I/R enhanced the expression levels of FGF-2, uPA, MMP-2, and MMP-9. IL-20 antibody injection hampered the induction of FGF-2, uPA, MMP-2, and MMP-9 (Figure 5A–E). It is well known that the most critical step in fibrotic scar formation is trans-differentiation of cardiac fibroblasts into myofibroblasts, we then examined and revealed that expression levels of alpha smooth muscle actin (α-SMA), a marker of myofibroblasts, were upregulated in I/R heart. IL-20 antibody treatment could reverse this phenomenon (Figure 5A,F). Besides determining the molecular changes, we further investigated the histological alterations in the ventricular tissues following I/R. Results from Masson’s Trichrome staining revealed excess accumulation of collagen in the ventricular tissues after I/R, and the injection of IL-20 antibody reduced collagen deposition (Figure 5G,H). In addition, we confirmed the expression levels of cardiac remodeling gene were elevated in anterior ventricular wall and IL-20 antibody treatment mitigated the expression levels of cardiac remodeling gene (Figure 5I). Moreover, we also revealed that the inhibition of IL-20, IL-20 antibody, DPI, and GSH attenuated H/R-upregulated mRNA expression level of FGF2, uPA, MMP-2, MMP-9, and α-SMA in H9C2 cells (Figure 5J–N).

### 3.6. Treatment with Anti-IL-20 Antibody Reduces Myocardial Infarction-Caused Heart Function Impairment

**Figure 6:**
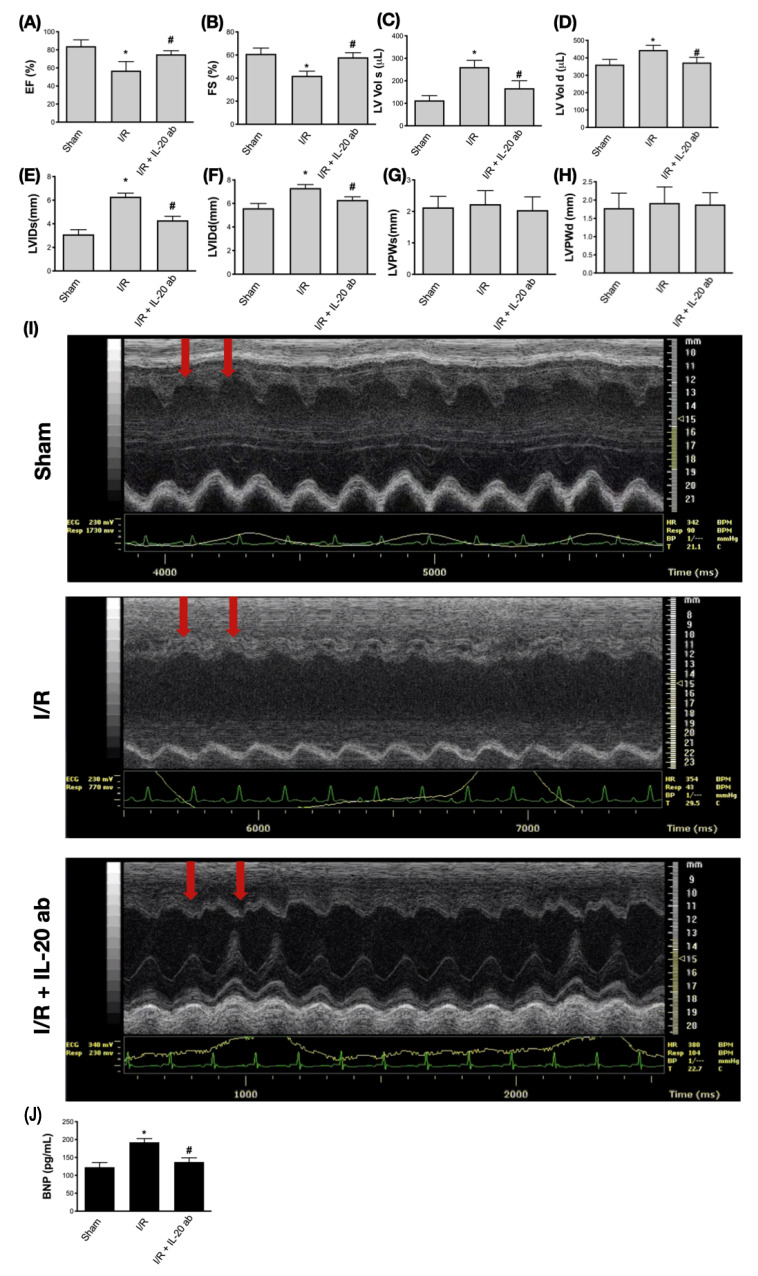
Examination of cardiac function using echocardiography. The parameters of the left ventricular function were measured using M-mode echocardiography. The ejection fraction (EF) (**A**) and fractional shortening (FS) (**B**) of left ventricle were measured to determine the left ventricular contraction ability. The volume of the left ventricle at end systole (**C**) and at end diastole (**D**), the internal dimension of left ventricle at end systole (**E**) and at end diastole (**F**), as well as the left ventricular posterior wall thickness at end systole (**G**) and at end diastole (**H**) were measured to determine the dilatation of left ventricle. Representative images of M-mode echocardiography were shown to demonstrate the rescue of ischemia/reperfusion (I/R)-induced cardiac dysfunction by interleukin (IL)-20 antibody treatment. Red arrows indicate the anterior wall of left ventricle (**I**). The concentrations of plasma B-type natriuretic peptide (BNP) were checked to determine the degree of cardiac dysfunction (**J**). The data were presented as the mean ± standard deviation of six animals in each group (* indicating *p* < 0.05 compared to the sham group; # indicating *p* < 0.05 compared to the I/R group).

**Figure 7:**
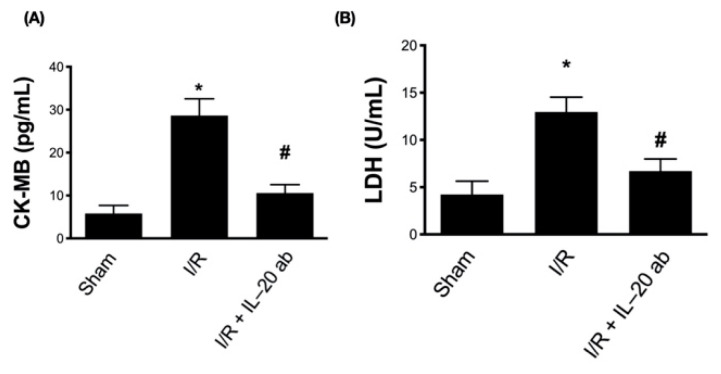
The effect of interleukin (IL)-20 antibody on the concentration of biomarkers for cardiac damage. The concentrations of creatine kinase-MB (CK-MB) (**A**) and lactate dehydrogenase (LDH) (**B**) were checked to determine the extent of myocardial infarction after ischemia/reperfusion (I/R) and the protective effect of IL-20 antibody treatment. The data were presented as the mean ± standard deviation of six animals in each group (* indicating *p* < 0.05 compared to the sham group; # indicating *p* < 0.05 compared to the I/R group).

Next, to evaluate the therapeutic effect of IL-20 antibody in myocardial infarction, we used echocardiography to monitor cardiac function. The left ventricular ejection fraction (Figure 6A) and fractional shortening (Figure 6B) were decreased in the setting of I/R, indicating that the contractive function of left ventricle was reduced by myocardial injury. Treatment with IL-20 antibody could improve the left ventricular contractive function in terms of ejection fraction (Figure 6A) and fractional shortening (Figure 6B). Myocardial injury caused dilatation of left ventricle, represented by the increased volume of left ventricle at either end systole (Figure 6C) or end diastole (Figure 6D) as well as increased internal dimension of left ventricle at both end systole (Figure 6E) and end diastole (Figure 6F). Injection of IL-20 antibody could reverse the effects of I/R injury on chamber dilatation of the left ventricle (Figure 6C–F). There was no significant difference in the thickness of the left ventricular posterior wall at end systole (Figure 6G) and end diastole (Figure 6H) among sham group, I/R group, and I/R plus IL-20 antibody treatment group, suggesting that the ligation of LAD coronary artery was successful without interfering other coronary artery flow. The function of the anterior wall of left ventricle was impaired whereas the posterior wall was unaffected. In addition, compared to control animals (Figure 6I, upper panel), the motion of the anterior wall of left ventricle (red arrows) in M-mode echocardiography was decreased under I/R injury (Figure 6I, middle panel). Treatment with IL-20 antibody improved the impaired motion of anterior wall (Figure 6I, lower panel). Impaired left ventricular contraction and dilatation of left ventricle are important features of CHF. Our data imply that IL-20 antibody has the ability to protect heart function from I/R injury. This finding was supported by plasma BNP concentration examination. The increase in plasma levels of BNP after I/R insult was attenuated by IL-20 antibody injection (Figure 6J). Moreover, in compliance with our previous results, we further proved that the concentrations of LDH and CK-MB in plasma were increased after I/R intervention. These two factors are commonly used to determine the severity of myocardial injury [35]. As expected, we confirmed that IL-20 antibody injection at reperfusion is an effective way to mitigate the I/R-induced myocardial injury (Figure 7A,B).

## 4. Discussion

**Figure 8:**
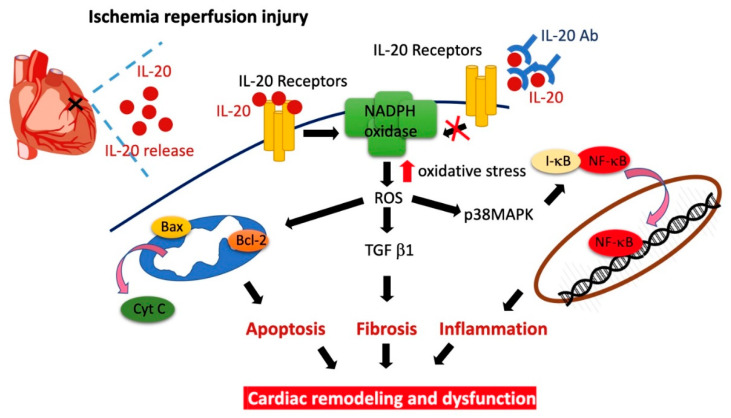
Schematic diagram of the major study findings.

Attenuating the ischemic period in the setting of AMI has attracted a considerable deal of attention owing to the strong relationship between the period of ischemia and the severity of myocardial damage. However, as soon as occlusion of the coronary artery is rebuilt, the myocardium is influenced by reperfusion injury [36]. Thus, it is important to develop new strategies to lessen I/R injury. The molecular mechanisms and biological signaling pathways regarding I/R damage are complex. The oxidative stress-induced myocardial injury is considered a critical regulator in the initiation of I/R damage [37]. The critical roles of oxidative injuries in I/R-caused myocardial remodeling have been determined by the investigation of extracellular matrix deposition, myocardial inflammation, fibrosis, and apoptosis. All of these events lead to development of CHF [38]. We previously demonstrated that IL-20 plays a key role in modulation of cardiac damage following myocardial infarction. In this study, we showed that IL-20 antibody injection at the moment of reperfusion reduces I/R-caused oxidative injuries, apoptosis, inflammation, as well as the elevation in markers of cardiac fibrosis and remodeling (Figure 8). We also demonstrated that IL-20 antibody injection attenuates cardiac functional impairment, including the repressed EF and FS, the increased left ventricular end-diastolic and end-systolic volumes, and downregulation of plasma markers of cardiac injury and heart failure. This study provides convincing evidence that IL-20 antibody is favorable in lessening reperfusion injury for treating AMI.

NADPH oxidase has been recognized as one of the main sources for ROS production in cardiomyocytes by transferring one electron to oxygen from NADPH [39]. A growing body of research has suggested that NADPH oxidase plays a critical role in controlling myocardial damage in I/R injury model. For example, a previous study revealed that the expression of NOX-2 was increased in rat ventricle after myocardial infarction [40]. This finding was further confirmed in human myocardial tissue. Krijnen et al. found a massive upregulation of NOX-2 expression in both viable and jeopardized cardiomyocytes in infarcted myocardial tissue [41]. The inhibition of NADPH oxidase activity decreased apoptosis in cardiomyocytes, prevented ventricular dysfunction, and increased survival rate in NOX-2 knockout animals following ischemic surgery [42]. In addition, NADPH oxidase has been suggested to be an important origin of mitochondrial superoxide. NADPH oxidase could be activated by ATP, indicating that NADPH oxidase can team mitochondrial oxidative stress-related signaling pathways [43]. Results from cardiac-specific NADPH oxidase-4 knockout animals have proved that NADPH oxidase contributes to mitochondrial impairment, myocardial apoptosis, and myocardial dysfunction by upregulating ROS [44]. In this study, we revealed that IL-20 antibody injection at the moment of reperfusion reduced the I/R-induced expression of NOX-2, Rac-1, and p47phox in myocardial tissue and elevation of oxidative stress. Moreover, IL-20 antibody treatment diminished I/R-induced expression of p-53, Bax, and cytochrome c, as well as restored the expression of Bcl-2. These results suggest that IL-20 antibody repressed I/R-caused myocardial apoptosis through modulation of oxidative stress and mitochondria-dependent apoptosis pathway.

The proinflammatory responses caused by I/R injury are one of the most important effects on the progression of myocardial impairment [45]. It has been reported that ROS overgeneration is responsible for the activation of the NF-κB signal pathway. Increased ROS can stimulate expression of NF-κB p65 subunit and its upstream signaling molecule IKK, both of which modulate the proinflammatory events [46]. In addition, a previous study suggested that early upregulation of NF-κB activity by I/R injury may be responsible for the regulation of immediate-early gene expression, providing an effective protection against myocardial ischemic injury [47]. In this study, we showed that IL-20 antibody suppressed the NF-κB pathway and IL-8 release, supporting the attenuation of I/R myocardial injury by IL-20 antibody is through blockade of inflammation.

Upregulation of TGF-β isoforms has been comprehensively confirmed in both animals with I/R injury and patients suffering from myocardial infarction [48]. Under the reperfusion of myocardial infarction, TGF-β1 has an early peak of mRNA expression at the time period of 6–72 h after reperfusion. In contrast, TGF-β3 mRNA expression is continually increased 7 days after reperfusion [49]. TGF-β is an upstream regulator of the MAPK pathway and essentially participated in the progression of myocardial repair and cardiac remodeling [30]. Moreover, repression of the MAPK pathway mitigated the pathological and molecular damage caused by I/R injury and ameliorated clinical outcomes in preclinical studies [50]. Here we showed that IL-20 antibody reduced the expression levels of TGF-β1 and p-ERK in rats experiencing I/R injury. Moreover, the I/R-mediated upregulation of Sp1, a critical regulator in controlling the expression of TGF-β family [51], was inhibited by IL-20 antibody injection. Since CTGF has also been involved in TGF-β1-triggered collagen synthesis in fibroblasts [52], we observed that CTGF expression was increased by I/R injury and IL-20 antibody injection impeded this upregulation as expected.

The cardiac remodeling is an advancing procedure affecting changes in cardiac hypertrophy, apoptosis of cardiomyocytes, proinflammatory responses, as well as cardiac fibrosis. Different remodeling responses may eventually cause impairment of cardiac function, thereby leading to heart failure [53]. FGF2, uPA, and MMPs all have been reported to be major regulators in the progression of cardiac repair and remodeling [34]. MMP-2 and MMP-9 are obviously expressed after AMI. In addition, MMP-9 knockout animals with I/R injury have less collagen accumulation in the infarcted area [32,54]. Our data showed that IL-20 antibody injection reduced the elevated expression of FGF2, uPA, MMP-2, and MMP-9 after I/R, which may ameliorate myocardial remodeling and cardiac functions.

This study has several limitations. First, the fibrotic scar formation and the cardiac remodeling process should take much more time to be completed. We did not test the long-term protective effects of IL-20 antibody in I/R model. Secondly, we did not quantify the amount of IL-20 antibody effectively reaching the cardiac tissue. Thirdly, we did not conduct double-stain with Evan’s blue and triphenyl tetrazolium chloride to detect the area of myocardium at ischemic risk caused by ligation of coronary artery in this study. These issues will be our major direction for further study.

## 5. Conclusions

In conclusion, this study uncovered that the profound protective effects of IL-20 antibody may be attributed to repression of NADPH oxidase-mediated oxidative stress and apoptosis of myocardium in I/R rats. Treatment with IL-20 antibody resulted in salutary effects on I/R-induced cardiac inflammation, fibrosis, and remodeling, thereby restoring cardiac function. Thus, the administration of IL-20 antibody in myocardial reperfusion, either by thrombolytic medication or using percutaneous coronary intervention, to repress myocardial ischemia-reperfusion injury could be a promising novel strategy for the improvement of AMI treatment.

## Data Availability

Not applicable.

## Supplementary Materials

The following are available online at https://www.mdpi.com/2076-3921/10/2/275/s1.

## References

[1] Lauridsen M.D., Rorth R., Lindholm M.G., Kjaergaard J., Schmidt M., Moller J.E., Hassager C., Torp-Pedersen C., Gislason G., Kober L. (2020). Trends in first-time hospitalization, management, and short-term mortality in acute myocardial infarction-related cardiogenic shock from 2005 to 2017: A nationwide cohort study. Am. Heart J..

[2] Zhang X., Wang S., Liu J., Wang Y., Cai H., Wang D., Fang S., Yu B. (2021). D-dimer and the incidence of heart failure and mortality after acute myocardial infarction. Heart.

[3] Mahowald M.K., Alqahtani F., Alkhouli M. (2020). Comparison of Outcomes of Coronary Revascularization for Acute Myocardial Infarction in Men Versus Women. Am. J. Cardiol..

[4] Smolders V.F., Zodda E., Quax P.H.A., Carini M., Barbera J.A., Thomson T.M., Tura-Ceide O., Cascante M. (2018). Metabolic Alterations in Cardiopulmonary Vascular Dysfunction. Front. Mol. Biosci..

[5] Bentzon J.F., Otsuka F., Virmani R., Falk E. (2014). Mechanisms of plaque formation and rupture. Circ. Res..

[6] Neri M., Riezzo I., Pascale N., Pomara C., Turillazzi E. (2017). Ischemia/Reperfusion Injury following Acute Myocardial Infarction: A Critical Issue for Clinicians and Forensic Pathologists. Mediat. Inflamm..

[7] Yellon D.M., Hausenloy D.J. (2007). Myocardial reperfusion injury. N. Engl. J. Med..

[8] Gonzalez-Montero J., Brito R., Gajardo A.I., Rodrigo R. (2018). Myocardial reperfusion injury and oxidative stress: Therapeutic opportunities. World J. Cardiol..

[9] Maejima Y., Kuroda J., Matsushima S., Ago T., Sadoshima J. (2011). Regulation of myocardial growth and death by NADPH oxidase. J. Mol. Cell. Cardiol..

[10] Hoffmeyer M.R., Jones S.P., Ross C.R., Sharp B., Grisham M.B., Laroux F.S., Stalker T.J., Scalia R., Lefer D.J. (2000). Myocardial ischemia/reperfusion injury in NADPH oxidase-deficient mice. Circ. Res..

[11] Ago T., Kuroda J., Pain J., Fu C., Li H., Sadoshima J. (2010). Upregulation of Nox4 by hypertrophic stimuli promotes apoptosis and mitochondrial dysfunction in cardiac myocytes. Circ. Res..

[12] Orogo A.M., Gustafsson A.B. (2013). Cell death in the myocardium: My heart won’t go on. IUBMB Life.

[13] Talman V., Ruskoaho H. (2016). Cardiac fibrosis in myocardial infarction-from repair and remodeling to regeneration. Cell Tissue Res..

[14] Yu X., Deng L., Wang D., Li N., Chen X., Cheng X., Yuan J., Gao X., Liao M., Wang M. (2012). Mechanism of TNF-alpha autocrine effects in hypoxic cardiomyocytes: Initiated by hypoxia inducible factor 1alpha, presented by exosomes. J. Mol. Cell. Cardiol..

[15] Wollert K.C., Drexler H. (2001). The role of interleukin-6 in the failing heart. Heart Fail. Rev..

[16] Prabhu S.D., Frangogiannis N.G. (2016). The Biological Basis for Cardiac Repair After Myocardial Infarction: From Inflammation to Fibrosis. Circ. Res..

[17] Hsu Y.H., Chang M.S. (2014). The therapeutic potential of anti-interleukin-20 monoclonal antibody. Cell Transplant..

[18] Tsai K.L., Hsieh P.L., Chou W.C., Hung C.H., Yang H.L., Chang Y.C., Chu P.M., Chang M.S., Chan S.H. (2020). IL-20 promotes hypoxia/reoxygenation-induced mitochondrial dysfunction and apoptosis in cardiomyocytes by upregulating oxidative stress by activating the PKC/NADPH oxidase pathway. Biochim. Biophys. Acta Mol. Basis Dis..

[19] Wei C.C., Hsu Y.H., Li H.H., Wang Y.C., Hsieh M.Y., Chen W.Y., Hsing C.H., Chang M.S. (2006). IL-20: Biological functions and clinical implications. J. Biomed. Sci..

[20] Hsu Y.H., Hsing C.H., Li C.F., Chan C.H., Chang M.C., Yan J.J., Chang M.S. (2012). Anti-IL-20 monoclonal antibody suppresses breast cancer progression and bone osteolysis in murine models. J. Immunol..

[21] Hsu Y.H., Chen W.Y., Chan C.H., Wu C.H., Sun Z.J., Chang M.S. (2011). Anti-IL-20 monoclonal antibody inhibits the differentiation of osteoclasts and protects against osteoporotic bone loss. J. Exp. Med..

[22] Matsushima S., Tsutsui H., Sadoshima J. (2014). Physiological and pathological functions of NADPH oxidases during myocardial ischemia-reperfusion. Trends Cardiovasc. Med..

[23] Dong Y., Chen H., Gao J., Liu Y., Li J., Wang J. (2019). Molecular machinery and interplay of apoptosis and autophagy in coronary heart disease. J. Mol. Cell. Cardiol..

[24] Levine A.J. (1997). p53, the cellular gatekeeper for growth and division. Cell.

[25] Ong S.B., Samangouei P., Kalkhoran S.B., Hausenloy D.J. (2015). The mitochondrial permeability transition pore and its role in myocardial ischemia reperfusion injury. J. Mol. Cell. Cardiol..

[26] Anversa P., Cheng W., Liu Y., Leri A., Redaelli G., Kajstura J. (1998). Apoptosis and myocardial infarction. Basic Res. Cardiol..

[27] Gottlieb R.A., Burleson K.O., Kloner R.A., Babior B.M., Engler R.L. (1994). Reperfusion injury induces apoptosis in rabbit cardiomyocytes. J. Clin. Invest..

[28] Saini H.K., Xu Y.J., Zhang M., Liu P.P., Kirshenbaum L.A., Dhalla N.S. (2005). Role of tumour necrosis factor-alpha and other cytokines in ischemia-reperfusion-induced injury in the heart. Exp. Clin. Cardiol..

[29] Ueha S., Shand F.H., Matsushima K. (2012). Cellular and molecular mechanisms of chronic inflammation-associated organ fibrosis. Front. Immunol..

[30] Bujak M., Frangogiannis N.G. (2007). The role of TGF-beta signaling in myocardial infarction and cardiac remodeling. Cardiovasc. Res..

[31] Xie L., Law B.K., Chytil A.M., Brown K.A., Aakre M.E., Moses H.L. (2004). Activation of the Erk pathway is required for TGF-beta1-induced EMT in vitro. Neoplasia.

[32] Yang H.L., Hsieh P.L., Hung C.H., Cheng H.C., Chou W.C., Chu P.M., Chang Y.C., Tsai K.L. (2020). Early Moderate Intensity Aerobic Exercise Intervention Prevents Doxorubicin-Caused Cardiac Dysfunction Through Inhibition of Cardiac Fibrosis and Inflammation. Cancers.

[33] Kardami E., Jiang Z.S., Jimenez S.K., Hirst C.J., Sheikh F., Zahradka P., Cattini P.A. (2004). Fibroblast growth factor 2 isoforms and cardiac hypertrophy. Cardiovasc. Res..

[34] Heymans S., Luttun A., Nuyens D., Theilmeier G., Creemers E., Moons L., Dyspersin G.D., Cleutjens J.P., Shipley M., Angellilo A. (1999). Inhibition of plasminogen activators or matrix metalloproteinases prevents cardiac rupture but impairs therapeutic angiogenesis and causes cardiac failure. Nat. Med..

[35] Hackel D.B., Reimer K.A., Ideker R.E., Mikat E.M., Hartwell T.D., Parker C.B., Braunwald E.B., Buja M., Gold H.K., Jaffe A.S. (1984). Comparison of enzymatic and anatomic estimates of myocardial infarct size in man. Circulation.

[36] Turer A.T., Hill J.A. (2010). Pathogenesis of myocardial ischemia-reperfusion injury and rationale for therapy. Am. J. Cardiol..

[37] De Vries D.K., Kortekaas K.A., Tsikas D., Wijermars L.G., van Noorden C.J., Suchy M.T., Cobbaert C.M., Klautz R.J., Schaapherder A.F., Lindeman J.H. (2013). Oxidative damage in clinical ischemia/reperfusion injury: A reappraisal. Antioxid. Redox Signal..

[38] Travers J.G., Kamal F.A., Robbins J., Yutzey K.E., Blaxall B.C. (2016). Cardiac Fibrosis: The Fibroblast Awakens. Circ Res.

[39] Bedard K., Krause K.H. (2007). The NOX family of ROS-generating NADPH oxidases: Physiology and pathophysiology. Physiol. Rev..

[40] Fukui T., Yoshiyama M., Hanatani A., Omura T., Yoshikawa J., Abe Y. (2001). Expression of p22-phox and gp91-phox, essential components of NADPH oxidase, increases after myocardial infarction. Biochem. Biophys. Res. Commun..

[41] Krijnen P.A., Meischl C., Hack C.E., Meijer C.J., Visser C.A., Roos D., Niessen H.W. (2003). Increased Nox2 expression in human cardiomyocytes after acute myocardial infarction. J. Clin. Pathol..

[42] Looi Y.H., Grieve D.J., Siva A., Walker S.J., Anilkumar N., Cave A.C., Marber M., Monaghan M.J., Shah A.M. (2008). Involvement of Nox2 NADPH oxidase in adverse cardiac remodeling after myocardial infarction. Hypertension.

[43] Shanmugasundaram K., Nayak B.K., Friedrichs W.E., Kaushik D., Rodriguez R., Block K. (2017). NOX4 functions as a mitochondrial energetic sensor coupling cancer metabolic reprogramming to drug resistance. Nat. Commun..

[44] Kuroda J., Ago T., Matsushima S., Zhai P., Schneider M.D., Sadoshima J. (2010). NADPH oxidase 4 (Nox4) is a major source of oxidative stress in the failing heart. Proc. Natl. Acad. Sci. USA.

[45] Xiong J., Xue F.S., Yuan Y.J., Wang Q., Liao X., Wang W.L. (2010). Cholinergic anti-inflammatory pathway: A possible approach to protect against myocardial ischemia reperfusion injury. Chin. Med. J. (Engl.).

[46] Dutot M., Liang H., Pauloin T., Brignole-Baudouin F., Baudouin C., Warnet J.M., Rat P. (2008). Effects of toxic cellular stresses and divalent cations on the human P2X7 cell death receptor. Mol. Vis..

[47] Li C., Browder W., Kao R.L. (1999). Early activation of transcription factor NF-kappaB during ischemia in perfused rat heart. Am. J. Physiol..

[48] Frangogiannis N.G. (2017). The role of transforming growth factor (TGF)-beta in the infarcted myocardium. J. Thorac. Dis..

[49] Dewald O., Ren G., Duerr G.D., Zoerlein M., Klemm C., Gersch C., Tincey S., Michael L.H., Entman M.L., Frangogiannis N.G. (2004). Of mice and dogs: Species-specific differences in the inflammatory response following myocardial infarction. Am. J. Pathol..

[50] Dobaczewski M., Chen W., Frangogiannis N.G. (2011). Transforming growth factor (TGF)-beta signaling in cardiac remodeling. J. Mol. Cell. Cardiol..

[51] Geiser A.G., Busam K.J., Kim S.J., Lafyatis R., O’Reilly M.A., Webbink R., Roberts A.B., Sporn M.B. (1993). Regulation of the transforming growth factor-beta 1 and -beta 3 promoters by transcription factor Sp1. Gene.

[52] Duncan M.R., Frazier K.S., Abramson S., Williams S., Klapper H., Huang X., Grotendorst G.R. (1999). Connective tissue growth factor mediates transforming growth factor beta-induced collagen synthesis: Down-regulation by cAMP. FASEB J..

[53] Abel E.D., Litwin S.E., Sweeney G. (2008). Cardiac remodeling in obesity. Physiol. Rev..

[54] Ducharme A., Frantz S., Aikawa M., Rabkin E., Lindsey M., Rohde L.E., Schoen F.J., Kelly R.A., Werb Z., Libby P. (2000). Targeted deletion of matrix metalloproteinase-9 attenuates left ventricular enlargement and collagen accumulation after experimental myocardial infarction. J. Clin. Invest..

