# Supplementary material for: Anti-IL-20 Antibody Protects against Ischemia/Reperfusion-Impaired Myocardial Function through Modulation of Oxidative Injuries, Inflammation and Cardiac Remodeling
Source: Antioxidants (Basel). 2021 Feb 10;10(2):275. doi: 10.3390/antiox10020275 (PMC7916786; doi:10.3390/antiox10020275)
Supplement: Supplementary file 1 [file antioxidants-10-00275-s001.pdf]

Supplementary Table S1. Primers of selected genes for quantitative real-time PCR

Gene	Primer sequences (5’-3’)
**β-actin**	F:5’-CCCTGGCTCCTAGCACCAT-3’
	R:5’-GATAGAGCCACCAATCCAATCCACACA-3’
**TGFβ1**	F:5’-CAAAGACATCACACACAGTA-3’
	R:5’- GGTGTTGAGCCCTTTCCAGG-3’
**SP1**	F:5’-GGCTACCCCTACCTCAAAGG-3’
	R:5’-CACAACATACTGCCCACCAG-3’
**CTGF**	F:5'-CAAGCTGCCCGGGAA AT-3'
	R:5'-CGGTCCTTGGGCTCATCA-3'
**FGF2**	F:5'-GAACCGGTACCTGGCTATGA-3'
	R:5'-CCGTTTTGGATCCGAGTTTA-3'
**uPA**	F:5'-CAGTCGAAGGCGGAACTCCTAT-3'
	R:5'-AGGCTAGGCCATTCTCTTCTC-3'
**MMP2**	F:5’- AAAGGAGGGCTGCATTGTGAA-3’
	R:5’-CTGGGGAAGGACGTGAAGAGG-3’
**MMP9**	F:5’-AGGTGCCTCGGATGGTTATCG-3’
	R:5’-TGCTTGCCCAGGAAGACGAA-3’
**α-SMA**	F:5’-GAGGCACCACTGAACCCTAA-3’
	R:5’- CATCTCCAGAGTCCAGCACA-3’
